# Transcriptomic profiling of *Alternaria longipes* invasion in tobacco reveals pathogenesis regulated by AlHK1, a group III histidine kinase

**DOI:** 10.1038/s41598-017-16401-6

**Published:** 2017-11-22

**Authors:** Juan Yang, Zhi-Qun Yin, Zi-Teng Kang, Chen-Jian Liu, Jin-Kui Yang, Jian-Hua Yao, Yi-Yong Luo

**Affiliations:** 1 Faculty of Life Science and Technology, Kunming University of Science and Technology, Kunming, 650500 China; 2 State Key Laboratory for Conservation and Utilization of Bio-Resources in Yunnan, Yunnan University, Kunming, 650091 China; 3 Yunnan Academy of Tobacco Science, Kunming, 650106 China

## Abstract

Tobacco brown spot, caused by *Alternaria* species, is a devastating tobacco disease. To explore the role of a group III histidine kinase (AlHK1) on *A. longipes* pathogenesis, the invasion progress of *A. longipes* was monitored. We found that the wild-type strain C-00 invaded faster than the *AlHK1*-disrupted strain HK∆4 in the early and middle infection stages and the reverse trend occurred in the late infection stage. Then, eight invasion transcriptomes were performed using RNA-Seq and 205 shared, 505 C-00 and 222 HK∆4 specific differentially expressed genes (DEGs) were identified. The annotation results showed seven antioxidant activity genes were specifically identified in the HKΔ4 DEGs. A subsequent experiment confirmed that HKΔ4 was more resistant to low concentrations oxidative stress than C-00. In addition, the results from 1) statistics for the number of DEGs, GO enriched terms, DEGs in clusters with rising trends, and 2) analyses of the expression patterns of some DEGs relevant for osmoadaptation and virulence showed that changes in C-00 infection existed mainly in the early and middle stages, while HKΔ4 infection arose mainly in the late stage. Our results reveal firstly the pathogenesis of *A. longipes* regulated by AlHK1 and provide useful insights into the fungal-plant interactions.

## Introduction

Phytopathogenic fungi in the genus *Alternaria* are a group of ubiquitous pathogenic fungi with necrotrophic lifestyle. *Alternaria* species infect a remarkable range of plants, including tobacco, citrus, and apple, among other species, which cause devastating plant disease around the world and result in considerable economic losses. In general, protective fungicides are widely used to control the crop diseases caused by *Alternaria* species. However, fungicide abuse can lead to environment pollution and a rapid rise in resistant strains. Therefore, a better understanding of the molecular interactions between *Alternaria* species and their hosts is necessary for developing alternative strategies to control *Alternaria* diseases.

As a necrotrophic pathogen, the pathogenesis of *Alternaria* species can be divided simply into two phases: first, the pathogen secretes effectors (phytotoxins, traditional proteinaceous effectors, etc.) to kill host cells or to induce programmed cell death, and then the dead tissues are degraded by various carbohydrate-active enzymes (CAZymes)^1^. In terms of host specificity, phytotoxins can be classified as host-selective toxins (HSTs) and non-host-selective toxins. Most HSTs are chemically diverse low-molecular-weight secondary metabolites and function as effectors in disease development^2^. So far, the bioactivities of 268 secondary metabolites from *Alternaria* species were reviewed^3^ and most of them were produced by polyketide synthase (PKS), non-ribosomal peptide synthase (NRPS), dimethylallyl tryptophan synthase (DMAT) and prenyltransferase (PT)/terpene synthase (TS)^4^. Therefore, mining PKS, NRPS, DMAT, PT/TS encoding genes or analysis of these gene expression profiles in the genome/transcriptome scale enable phytopathologists to easily evaluate the contribution of secondary metabolites to plant pathogenic fungi pathogenicity.

In the field of plant-microbe interactions, the term “effector” is widely used and defined as all pathogen-secreted proteins and small molecules that cause an effect on the host cell^5^. However, pathogen effectors, in a more narrow definition (i.e., traditional proteinaceous effectors), are cysteine-rich, small secreted proteins and they act on the plant innate immune system in a gene-for-gene manner that results in either suppression or development of disease^6^. In addition, accumulating evidence has shown that many secreted proteins (e.g., traditional proteinaceous effectors) are involved in pathogenic fungi pathogenesis^6,7^. Therefore, prediction and analysis of the secreted proteins and effectors from genome and transcriptome data are routinely reported in the field of plant-microbe interactions. For example, Dong *et al*. identified 134 candidate effectors in the *Magnaporthe oryzae* genome and two of them were involved in fungal pathogenesis through suppression of host defence-related gene expression^8^.

After host cells are killed by necrotrophic fungi, the next step for pathogen infection is to decompose the dead tissues with various CAZymes. CAZymes are an important class of proteins that are involved in the metabolism of glycoconjugates, oligo- and poly-saccharides. For plant pathogens, CAZymes are also related to the breakdown of the host cell wall. According to catalytic or catalysis-related modules, CAZymes are grouped into six classes: glycoside hydrolases (GHs), glycosyltransferases (GTs), polysaccharide lyases (PLs), carbohydrate esterases (CEs), auxiliary activities (AAs) and carbohydrate-binding modules (CBMs)^9^. Identifying and comparing CAZymes from different pathogenic fungi may provide additional information for a better understanding of their infection models. Most recently, many attempts to explore virulence factors from the CAZymes database have been conducted. For instance, Cho *et al*. reported that a pectate lyase, one of the PLs, was identified as a virulence factor among 106 differentially expressed genes (DEGs) regulated by the key-pathogenesis regulator AbPf2^10^.

Tobacco brown spot, caused by *A. longipes* or *A. alternata*, is one of the most serious leaf spot diseases, which limits tobacco planting and production. Similar to other necrotrophic plant pathogenic fungi, phytotoxin is one of the conventional weapons and plays an important role in the infection of *A. longipes* and *A. alternate* in tobacco. For example, AT-toxin, one of the HSTs, has been shown to be involved in the onset of tobacco brown spot^11^. In addition to phytotoxins, other virulence or virulence-related factors and pathways in *Alternaria* species, such as metabolite-associated effective detoxification of reactive oxygen species (ROS)^12,13^, transcription factors^14–16^, CAZymes^10^, two-component histidine kinase (HK)-mediated signalling pathways^17,18^, and a mitogen-activated protein kinase-mediated signalling pathway^19^ have been identified to date. In our previous study, a group III HK (AlHK1) was found to regulate *A. longipes* pathogenicity in a negative manner^18^.

Recently, transcriptome analysis has been widely adopted to explore the molecular interactions between phytopathogenic fungi and their hosts. However, most studies in this field have been focused on the host transcriptome of different plant cultivars in response to different pathogens. For example, the only transcriptome study of tobacco brown spot was to investigate the transcriptomic profiling of tobacco in response to *A. longipes* and *A. alternata* infection^20^. To determine the pathogenic mechanisms of *A. longipes* and the regulatory role of AlHK1 in this process, the infection process of *A. longipes* was first observed by confocal microscopy; then, the time-course invasion transcriptomes of a wild-type strain C-00 (hereinafter referred as C-00) and an *AlHK1*-disrupted strain HK∆4 (hereinafter referred as HKΔ4) were undertaken using high-throughput RNA sequencing (RNA-Seq).

## Results

### AlHK1 mediates *A. longipes* pathogenicity and invasion progress

**Figure 1:**
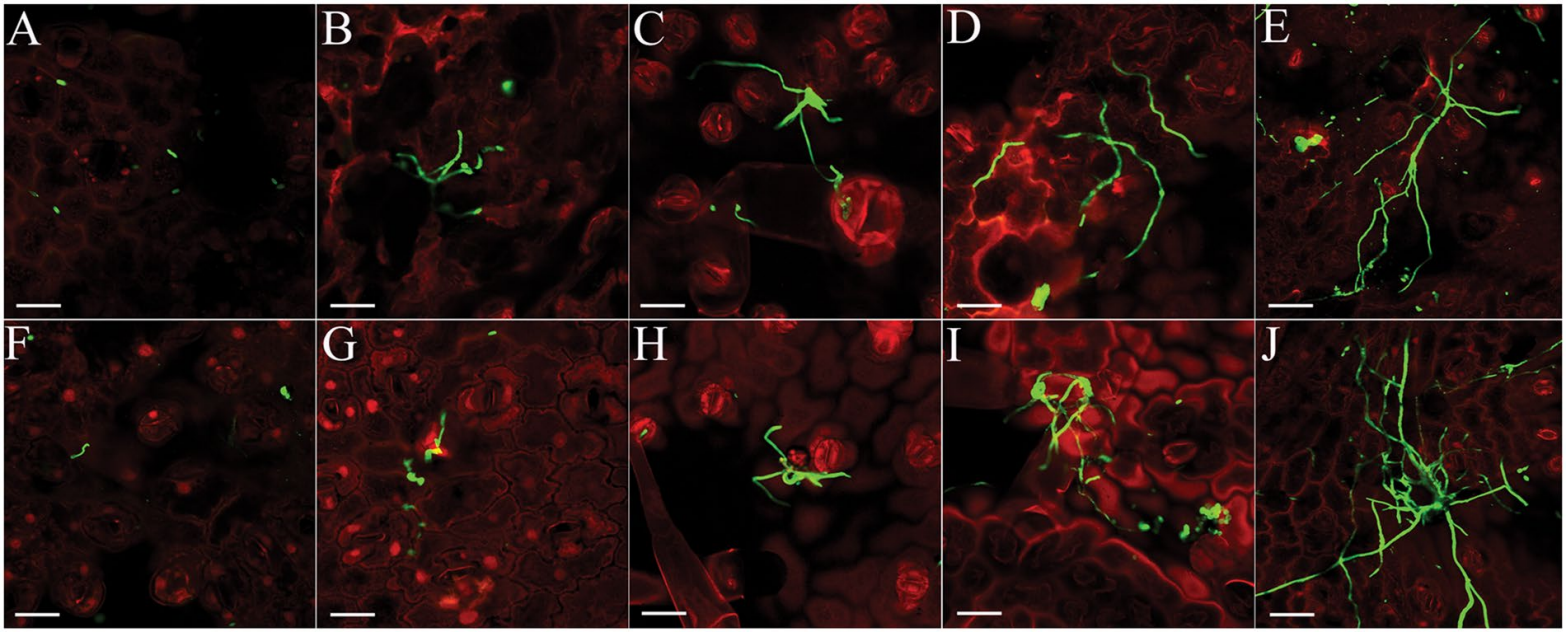
Confocal microscopy images during *A. longipes* infection. (**A–E**) C-00 and (**F–J**) HKΔ4 were incubated on the surfaces of detached tobacco leaves for 4, 6, 12, 20 and 24 hpi, respectively. Fungal material was stained with WGA-CF488A (green). Plant material was stained with propidium iodide (red). Bars: 100 µm. Only representative replicates are shown.

The involvement of the *AlHK1* gene in pathogenicity was evaluated using detached tobacco leaves inoculated with mycelial plugs. Similar to the pathogenic results inoculated with conidia^18^, both C-00 and HKΔ4 can produce obvious lesions, while the lesion of HKΔ4 was bigger compared to C-00 (Supplementary Fig. 1), although leaf symptom was not observed in the first two days of infection, which further supports the conclusion that AlHK1 negatively impacts *A. longipes* pathogenicity. To investigate this phenomenon in detail, the invasion progress of lesion-not-forming stage was observed using a confocal microscope. As shown in Fig. 1, C-00 and HKΔ4 had the same infection trend: the fungi began to invade at approximately 4 hours post-inoculation (hpi), the mycelial branches were formed at 6 hpi and then many branched hyphae expanded into their neighbouring plant tissues at 12 hpi. At and after 20 hpi, a branched multicellular network was formed, and the invasion is considered to be finished. Therefore, we determined ≤6 hpi, 7–19 hpi and ≥20 hpi as early, middle and late infection stage, respectively. Noteworthily, the mycelial invasion speed of C-00 was faster compared to HKΔ4 in the early and middle infection stage, while the situation was reversed in the late stage (Fig. 1). This trend subsequently results in HKΔ4 showing bigger lesions than C-00. These results indicate that AlHK1 may regulate *A. longipes* pathogenicity through a come-from-behind mechanism.

### Sequencing, *de novo* assembly and annotation of *A. longipes* transcriptome

Based on the microscopic observation, we sought to uncover the molecular basis underlying *A. longipes* pathogenesis using omics technology. Therefore, RNA-Seq libraries of C-00 and HKΔ4 from 0, 6, 12 and 20 hpi were constructed, sequenced with an Illumina/Solexa high-throughput sequencing platform, and the transcriptome profiling of C-00 and HKΔ4 during an invasion were obtained (Table 1). After removing substandard reads, a total of approximately 192 million 100 bp paired-end clean reads (98.03% of raw reads) were generated from 8 RNA-Seq libraries. The clean reads were *de novo* assembled, yielding 22,435 unique genes with a mean length of 1,314 bp and with a N50 in a length of 2,383 bp. For unigene annotation, 13,866, 11,104, 9,151 and 4,669 unigenes were annotated in Non-redundant (Nr), Swiss-Prot, and Cluster of Orthologous Groups (COG) and Kyoto Encyclopaedia of Genes and Genomes (KEGG) databases based on sequence homologies, which yielded 16,657 annotated unigenes (74.25% of all unigenes) in total. The results suggest that the quality of the data is high enough to conduct downstream analyses.

**Table 1:** Summary of Illumina sequencing and transcriptome assemblies for RNA-Seq libraries.

Sample	Clean reads(ratio)	Nucleotides (nt)	Mapped reads (ratio)
C-00 0 hpi	21,589,652 (97.76%)	2,698,706,500	18,712,425 (86.67%)
C-00 6 hpi	26,204,502 (98.21%)	3,275,562,750	19,266,771 (73.52%)
C-00 12 hpi	25,129,686 (98.16%)	3,141,210,750	18,419,541 (73.30%)
C-00 20 hpi	24,676,514 (98.23%)	3,084,564,250	17,765,576 (71.99%)
HKΔ4 0 hpi	25,593,108 (97.97%)	3,199,138,500	20,933,206 (81.79%)
HKΔ4 6 hpi	23,333,938 (97.86%)	2,916,742,250	19,551,598 (83.79%)
HKΔ4 12 hpi	22,159,388 (98.06%)	2,769,923,500	19,341,040 (87.28%)
HKΔ4 20 hpi	23,345,520 (97.96%)	2,918,190,000	18,435,407 (78.97%)
*A. longipes*	Unigene number	N50 (bp)	Average length (bp)
	22,435	2,383	1,314

### Analysis of differential gene expression during *A. longipes* infection

**Figure 2:**
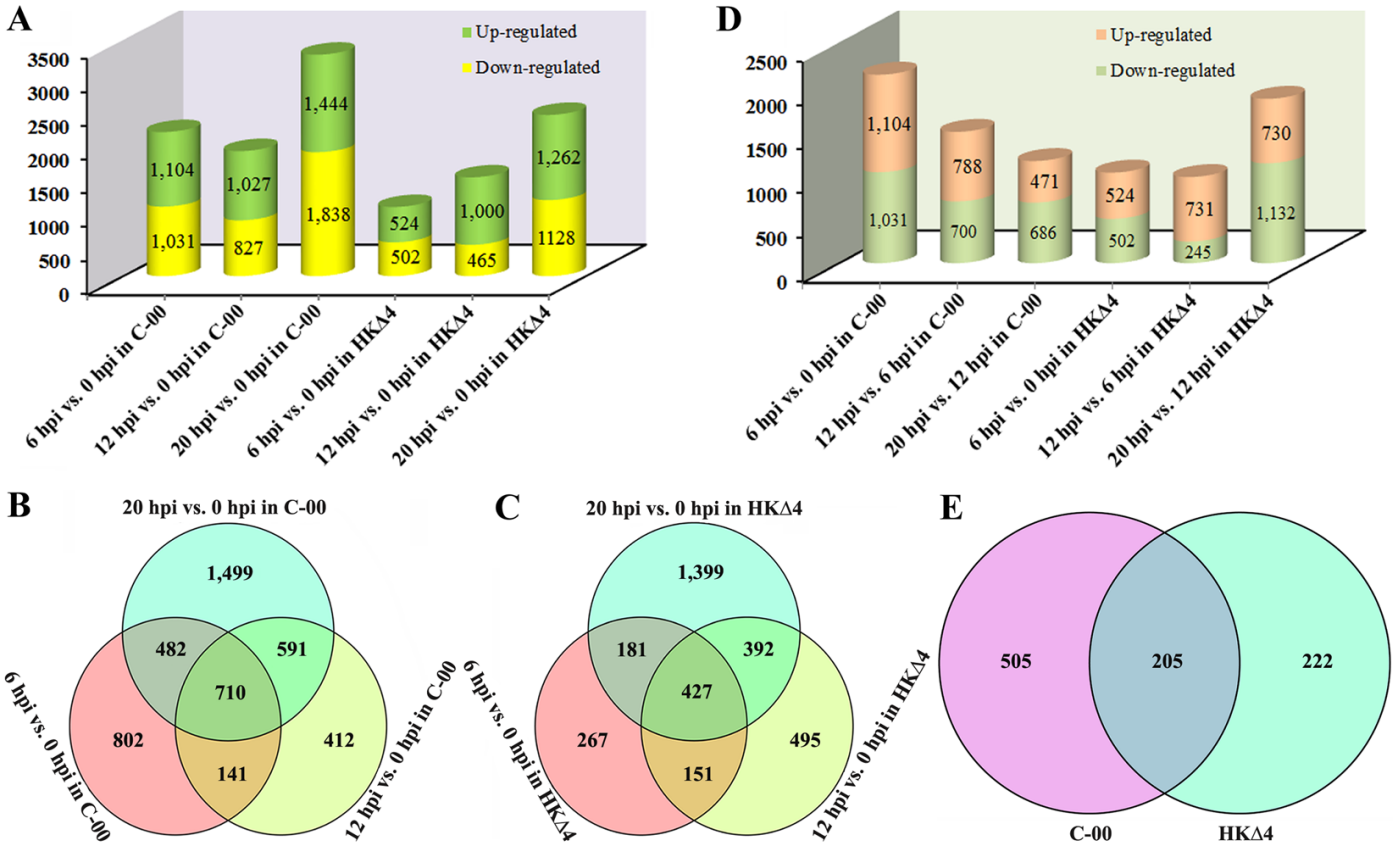
Summary for the DEGs. (**A**) Statistics of the number of DEGs at three infection time points compared to 0 hpi. (**B–C**) Venn diagram showing the number of shared and specific DEGs during C-00 and HKΔ4 infection, respectively. (**D**) Statistics of the number of DEGs between two adjacent time points. (**E**) Venn diagram showing the commonalities and differences between the shared DEGs of C-00 infection and the shared DEGs of HKΔ4 infection.

RPKM (reads per kilobase per million mapped reads) values were used to denote unigene expression level and DEGs were identified according to the criteria described in methods. Compared to 0 hpi, there were 2,135, 1,854 and 3,282 DEGs at 6, 12 and 20 hpi (referred as the three infection time points from this point on) in C-00 invasion progress, respectively (Fig. 2A). These DEGs were combined and a total of 4,637 DEGs including 710 shared unigenes were obtained (Fig. 2B). In comparison, the number of DEGs for HKΔ4 infection increased from 1,026 at 6 hpi, to 1,465 at 12 hpi, and finally to 2,390 at 20 hpi (Fig. 2A). Additionally, 3,303 DEGs were identified in total, from which 427 genes were shared DEGs (Fig. 2C). To compare the DEGs counts between C-00 and HKΔ4 infection, the ratio of DEGs in 6, 12 and 20 hpi to total DEGs was respectively calculated. The combined ratio of DEGs at 6 and 12 hpi in the C-00 infection was 80.62%, which had 5.21% more than that in the HKΔ4 infection, while the ratio of DEGs at 20 hpi in the C-00 infection (70.78%) was slightly smaller than that in the HKΔ4 infection (72.36%). The results indicate that changes of DEGs counts in the C-00 infection existed mainly in the early and middle stages (≤19 hpi), but HKΔ4 infection happened mostly in the late stage (≥20 hpi), which perfectly matched the *A. longipes* infection phenotype observed using confocal microscopy (Fig. 1). This indication is further supported by a statistics analysis for the number of DEGs between two adjacent time points. For C-00 infection, the number of DEGs (2,135) was the largest at 6 hpi vs. 0 hpi, which decreased to 1,488 at 12 hpi vs. 6 hpi, and finally to 1,157 at 20 hpi vs. 12 hpi (Fig. 2D). However, the situation for HKΔ4 infection was converse. The number of DEGs at 6 hpi vs. 0 hpi was 1,026, which slightly decreased (976) at 12 hpi vs. 6 hpi and sharply increased (1,862) at 20 hpi vs. 12 hpi (Fig. 2D).

### Shared and specific differentially expressed genes during *A. longipes* infection

**Figure 3:**
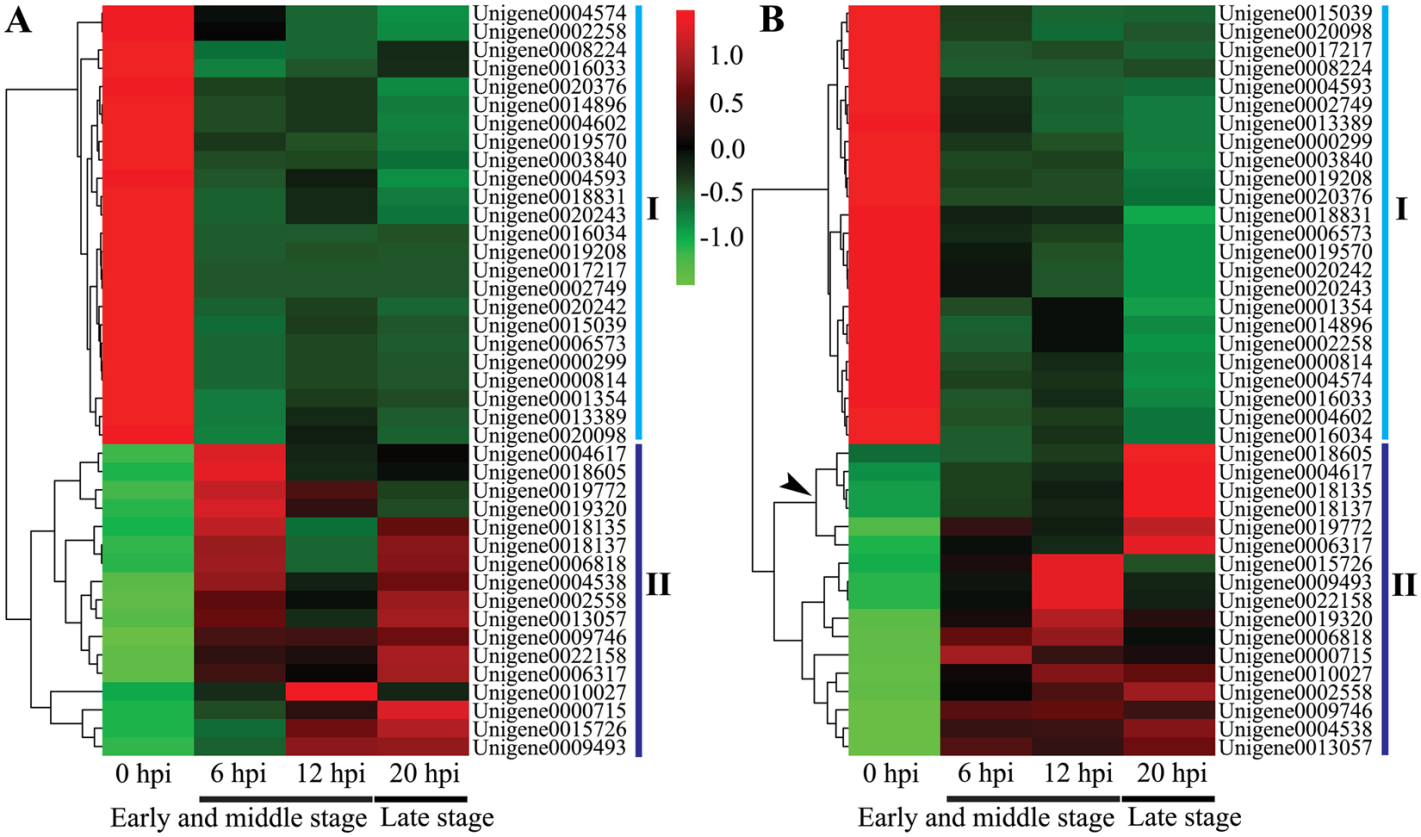
Heatmap showing the expression abundance of the prevalent shared DEGs encoding peptidases, transporters, cytochrome P450s and transcription factors during C-00 (**A**) and HKΔ4 (**B**) infection. The down-regulated and up-regulated genes (Supplementary Table 1) are shown with I and II, respectively. The colours correspond to the value of RPKM, which range from green (low expression) to red (high expression). The arrow indicates the six up-regulated genes whose expression abundance has a bias at 20 hpi.

The above mentioned two sets of shared DEGs during C-00 and HKΔ4 infection (Fig. 2B and C) were further analysed to find commonalities and differences. As shown in the Venn diagram of Fig. 2E, 205 shared as well as 505 C-00 and 222 HKΔ4 specific DEGs were identified. Of the 205 shared DEGs, 111 up-regulated and 89 down-regulated unigenes had the same trend between C-00 and HKΔ4 infection, while the remaining five unigenes showed different trends (Supplementary Table 1). According to the annotation, there were 15 peptidases, 13 transporters, seven cytochrome P450s and six transcription factors, which dominated the top 4 places. The data suggests that these prevalent genes may play conserved roles in *A. longipes* pathogenicity. Furthermore, the genes expression abundance of the prevalent shared DEGs was analysed over time using a heatmap. For C-00 infection, 24 unigenes showed similar RPKM values at 6 and 20 hpi that seemed smaller compared to the RPKM value at 12 hpi, while 17 up-regulated unigenes showed different expression patterns in which the greatest expression abundance appeared mainly at 6 and 12 hpi (the early and middle infection stages) (Fig. 3A). In contrast, the expression of unigenes had bias in the late stage (20 hpi) for HKΔ4 infection that was represented by all of the down-regulated unigenes and six up-regulated unigenes (Fig. 3B). Of the 505 specific DEGs in C-00 infection, 225 were up-regulated, 274 were down-regulated, and six exhibited fluctuating trends (Supplementary Table 1). In comparison, there were 93 up-regulated, 111 down-regulated and 18 unigenes with fluctuating trends in the HKΔ4 infection (Supplementary Table 1). Surprisingly, seven unigenes that show antioxidant activity (i.e., catalase, peroxidase and peroxiredoxin), in particular, two peroxidase genes that were up-regulated, were found in HKΔ4 specific DEGs, while none of these genes were among the C-00 specific DEGs (Supplementary Table 1). Together, the results from the analysis of expression abundance of the prevalent shared DEGs and from the identification of the specific DEGs between C-00 and HKΔ4 infection likely reveals partial reasons for why C-00 shows bigger a quicker invasion speed at the early and middle infection stages, while HKΔ4 shows stronger pathogenicity at the late infection stage.

### C-00 is more sensitive to low concentrations oxidative stress than HKΔ4 *in vitro*

**Figure 4:**
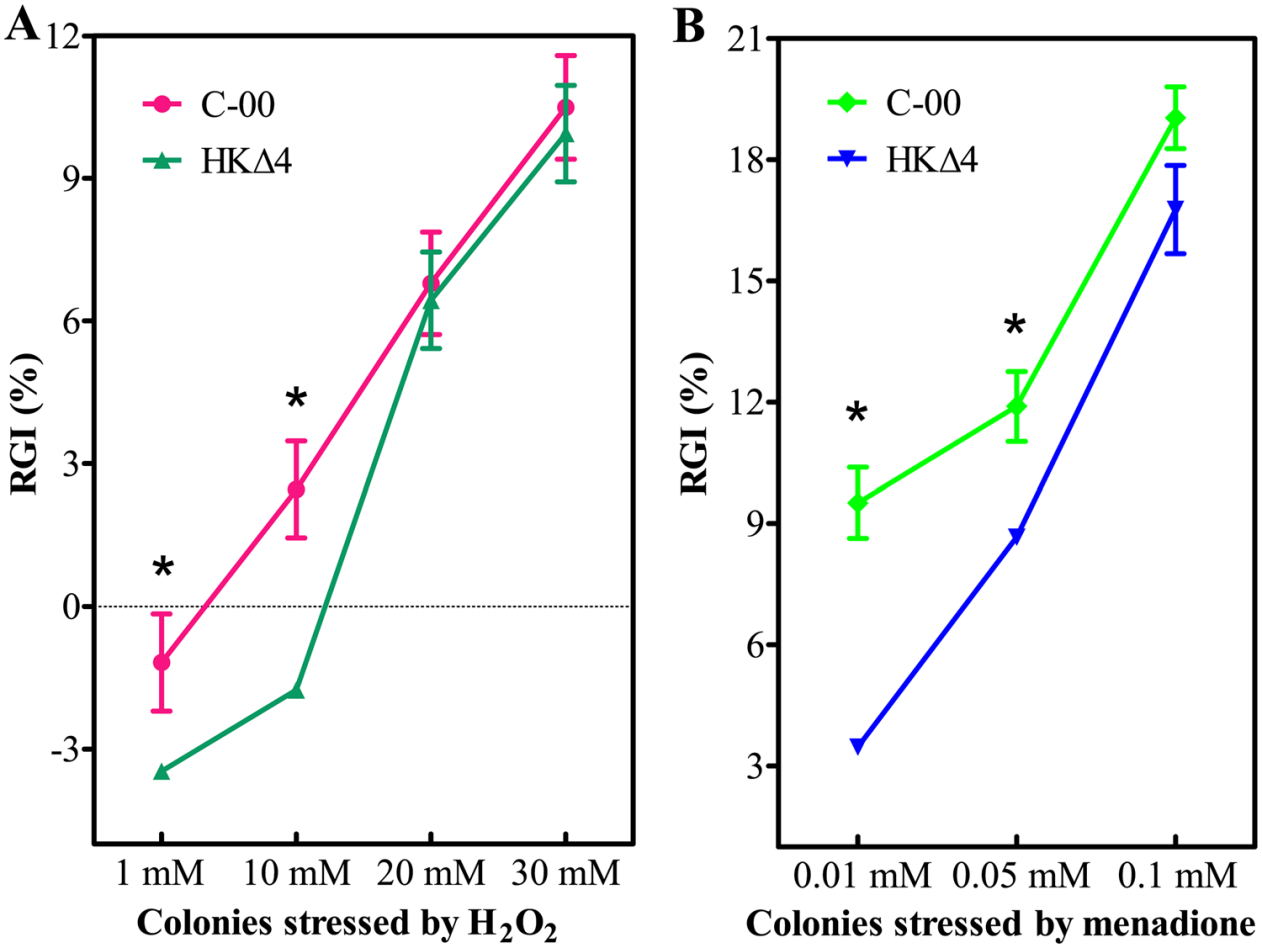
Effects of oxidative stress on hyphal growth. Error bars denote the standard deviations (SD) from three repeated experiments. Asterisks indicate a significant difference (*P* < 0.05) between strains C-00 and HKΔ4 at the same infection time point.

To test the hypothesis that some unigenes with antioxidant activity affect *A. longipes* virulence, the sensitivity of C-00 and HKΔ4 in response to oxidative stress was evaluated using hyphal growth assay. As shown in Fig. 4, the relative growth inhibition (RGI) was increased with an increase in the H_2_O_2_ and menadione concentration. The RGIs of C-00 were significantly larger compared to HKΔ4 when they were treated with 1 and 10 mM H_2_O_2_ or 0.01 and 0.05 mM menadione (*P* < 0.05). However, no significant difference (*P* > 0.05) was found under the situation with treatments of 30 mM H_2_O_2_ and 0.1 mM menadione (Fig. 4). The results indicate that C-00 is much more sensitive to low concentrations oxidative stress than HKΔ4, and AlHK1 likely mediates this process.

### Gene ontology (GO) enrichment analysis of differentially expressed genes during *A. longipes* infection

The DEGs obtained from three infection time points compared to 0 hpi were classified into various sub-categories according to their common characteristics or functions based on the GO classification system (http://geneontology.org/). For C-00 infection, six, six, and five enriched GO terms were identified at 6, 12 and 20 hpi, respectively, in which intrinsic to membrane (GO:0031224), membrane part (GO:0044425), membrane (GO:0016020), protein processing (GO:0016485) and protein maturation (GO:0051604) were shared enriched terms (Supplementary Table 2). The five shared enriched terms also existed in the HKΔ4 infection at 12 and 20 hpi, which suggests that they probably play a conserved role in *A. longipes* infection. As for the specific enriched terms, except for transporter activity (GO:0005215) and antioxidant activity (GO:0016209) that respectively uniquely exist in C-00 and HKΔ4 infection at 6 hpi, the C-00 infection was associated with two more terms (GO:0016485 and GO:0051604) than the HKΔ4 infection at 6 hpi (Supplementary Table 2). At 12 and 20 hpi, the terms and their counts for C-00 infection had hardly any changes (Supplementary Table 2). In contrast, the number of specific enriched terms for HKΔ4 infection began to increase from three terms that possessed peptidase activity (GO:0008233, GO:0004175 and GO:0070011) at 12 hpi to 11 terms that mainly included various transporters at 20 hpi (Supplementary Table 2). Thus, the data shows that the infection differential characteristics for C-00 and HKΔ4 are also reflected by GO enrichment analysis.

### Cluster of time-course RNA-Seq data

**Figure 5:**
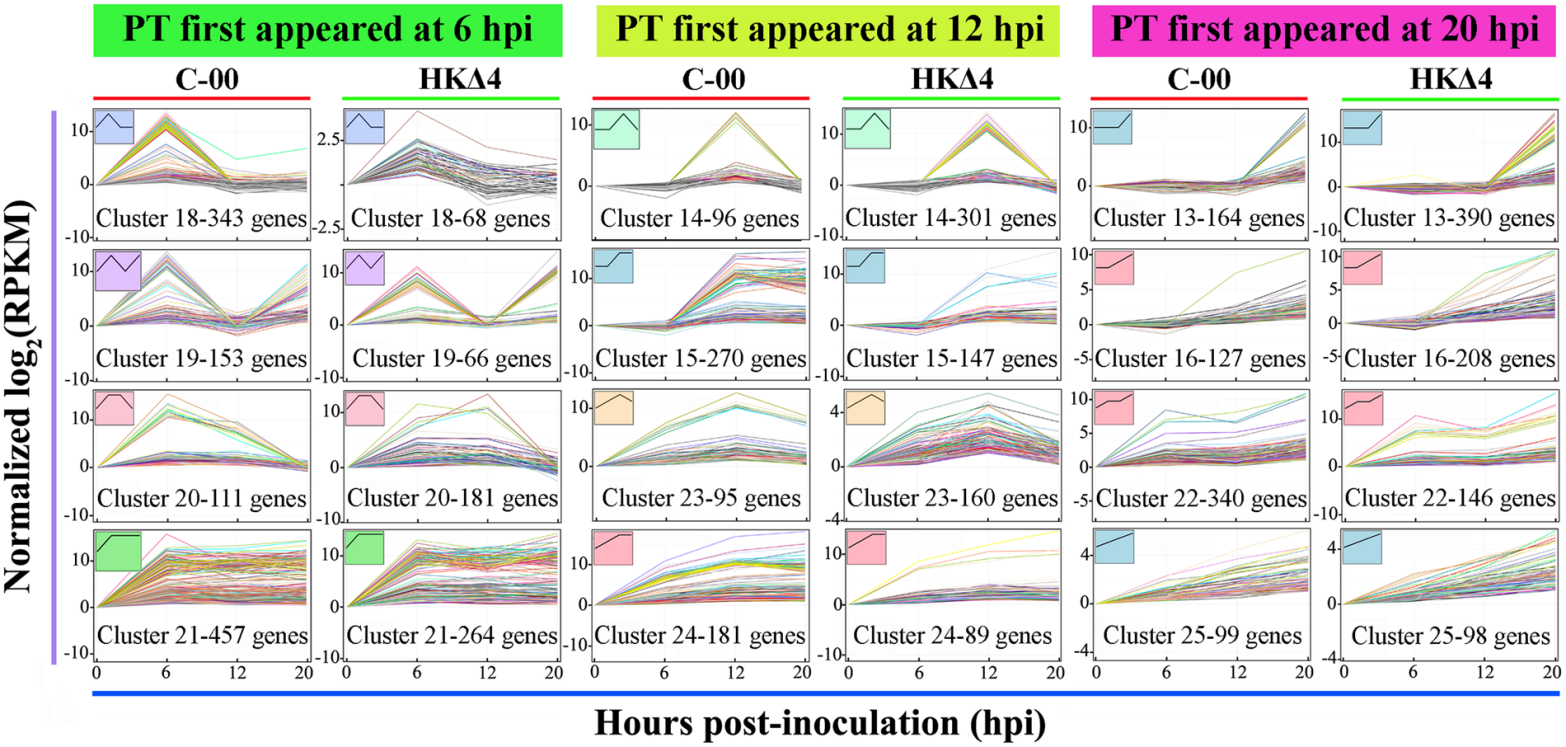
The DEGs clusters with rising trends. The clusters are characterized into three types: 1) peak transcription (PT) first appeared at 6 hpi, 2) PT first appeared at 12 hpi and 3) PT first appeared at 20 hpi. The number of DEGs is indicated in the bottom of each cell, and the trend line in the top left corner of each cell represents the DEGs trend.

Genes with similar expression patterns might be functionally correlated^21^. Using the short time series expression miner (STEM) algorithms, 26 significant clusters (*P* < 0.001) in C-00 or HKΔ4 infection based on the DEGs expression patterns similarity were identified, in which 12 clusters have rising trends (Fig. 5). Clusters 18, 19, 20 and 21 with peak transcription that first appeared at 6 hpi contained 1,064 DEGs in C-00 and 579 DEGs in HKΔ4 (Fig. 5). In comparison, there were similar counts of DEGs between C-00 (642) and HKΔ4 (692) in clusters 14, 15, 23 and 24 with a peak transcription that first appeared at 12 hpi (Fig. 5). In contrast, the number of DEGs of clusters 13, 16, 22 and 25 with a peak transcription that first appeared at 20 hpi in HKΔ4 was 842, which was bigger compared to C-00 (730 unigenes) (Fig. 5). This difference was highly consistent with the phenotypic difference between C-00 and HKΔ4 invasion progress, suggesting that an easier establishment of *A. longipes* infection (Fig. 1) may lead to an earlier transcriptomic response in C-00 compared to HKΔ4, and HKΔ4 shows more DEGs in the late infection stage (≥20 hpi) to make it catch up and overtake C-00 in the invasion progress.

### Expression pattern analyses of differentially expressed genes for fungal adaptation to osmotic stress

**Figure 6:**
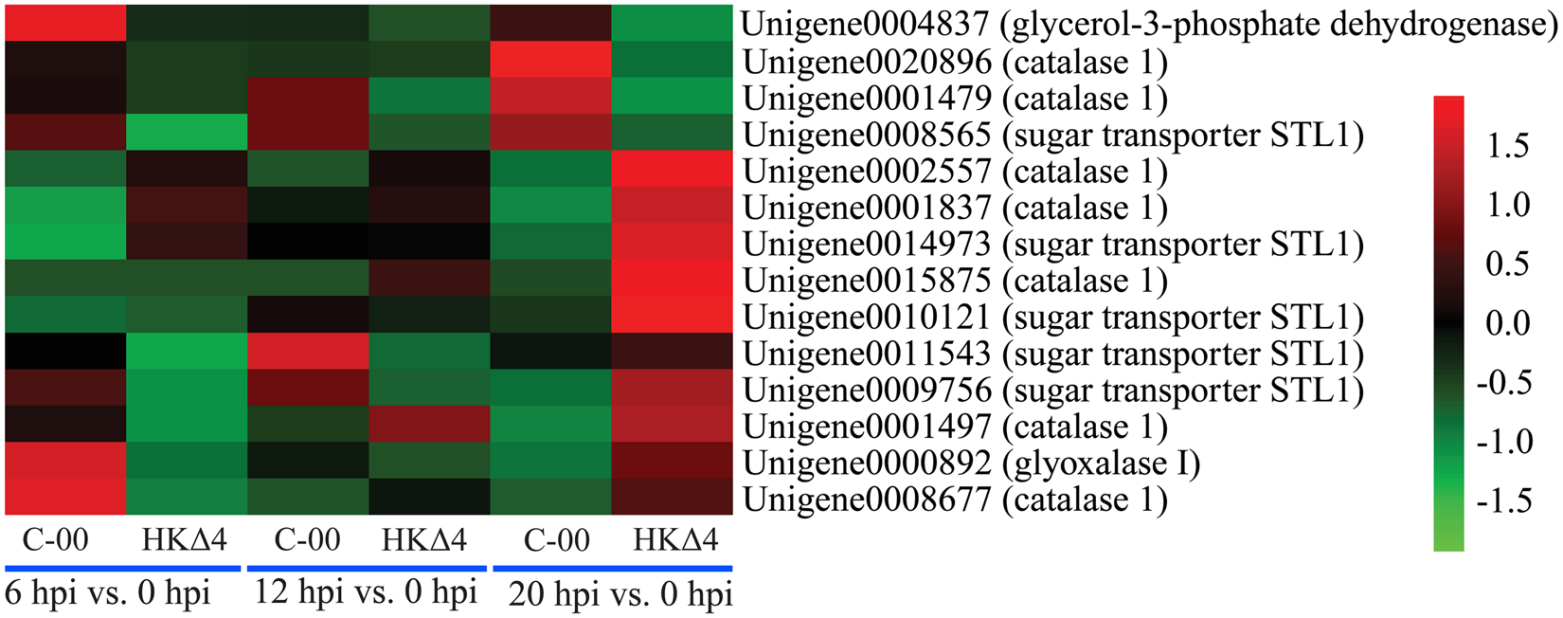
The expression patterns of osmoresponsive genes. The colours correspond to the ratio of RPKM at three infection time points to 0 hpi, which range from green (low expression) to red (high expression).

The high osmolarity glycerol (HOG) pathway mediates fungi adaptive response to osmotic stress by production of glycerol as a compatible osmolyte^22^. A small subset of proteins including sugar transporter STL1, glycerol-3-phosphate dehydrogenase and glycerol-3-phosphatase directly affects cytosolic glycerol concentrations^22^. In addition, the up-regulated expression of two stress sensor proteins (glyoxalase I and catalase 1) are also regulated by Hog1 mitogen-activated protein kinase under highly osmotic stress conditions^22,23^. Two-component system, serving as an upstream regulating pathway of the HOG signalling cascade, is involved in the high osmotic stress adaptation^22,24^. Therefore these osmoresponsive genes were selected according to the criteria of transcript level at one of three infection time points relative to 0 hpi being differentially expressed and their expression profiles were analysed. As shown in Fig. 6, four up-regulated genes (unigene0004837, unigene0000892, unigene0008677, unigene0009756) and two down-regulated genes (unigene0001837, unigene0014973) were identified at 6 hpi vs. 0 hpi for C-00 infection, whereas corresponding number for HKΔ4 infection was zero and four. The number of up- and down-regulated genes was similar between C-00 and HKΔ4 at 12 hpi vs. 0 hpi. Expectedly, HKΔ4 has more up-regulated genes than C-00 at 20 hpi vs. 0 hpi. The data suggests that C-00 may have an osmotic stress adaptive advantage in the early infection stage, while HKΔ4 may has the similar advantage in the late infection stage.

### Number and expression patterns of the secondary metabolite biosynthesis genes

Fungal pathogens generally produce a set of secondary metabolites, and some are involved in pathogenesis^2^. Using a SMURF (secondary metabolite unique regions finder) prediction, 22 secondary metabolite biosynthesis genes, including eight PKSs, 13 NRPSs and one DMAT, were identified (Supplementary Table 3). Among these genes, 14 and 10 DEGs, including nine shared, five C-00 and one HKΔ4 specific DEGs, were identified. Next, their expression patterns were determined and the change degree (CD) of expression level was calculated by the formula: CD = RPKM value at each of three infection time points - RPKM value at 0 hpi. The results showed that five (55.56%) out of the nine shared DEGs had the biggest change degree (BCD) at 6 and 12 hpi in the C-00 infection, while the BCD at 20 hpi had a 66.67% proportion in the HKΔ4 infection (Supplementary Fig. 2). For expression patterns of the specific DEGs, both C-00 and HKΔ4 DEGs (100%) showed the BCD at 6 and 12 hpi, suggesting that their expression level was not relevant to C-00 and HKΔ4 differential pathogenicity.

### Number and expression patterns of the secreted protein encoding genes

Accumulating evidence reveals a strong relationship between secreted proteins in fungal genomes and infection success^7^. According to prediction algorithms described in methods, 644 potential secreted proteins containing 188 shared, 106 C-00 and 56 HKΔ4 specific DEGs were identified (Supplementary Fig. 3). Among the shared DEGs, there were 38, 20 and 130 unigenes that showed the BCD at C-00 6, 12 and 20 hpi, respectively. In comparison, the corresponding number in the HKΔ4 infection was 25, 30 and 133 (Supplementary Fig. 3). As for the specific DEGs, 42 (39.62%) and 19 (33.93%) out of the corresponding specific DEGs exhibited the BCD at 6 and 12 hpi in C-00 and HKΔ4 infection, respectively (Supplementary Fig. 3). The results from the analyses of expression patterns of the shared or the specific DEGs display a bit more DEGs that show a BCD at 6 and 12 hpi and a slightly fewer DEGs that show a BCD at 20 hpi in the C-00 infection compared to the HKΔ4 infection.

### Number and expression patterns of the candidate effectors encoding genes

Effectors of fungal pathogens are involved in recognition by their host cells and play important roles in their pathogenicity^6^. After screening from the secreted proteins repertoire, 82 unigenes with intact transcripts of a length of less than 200 amino acids and with a cysteine content of at least 1.5% were considered to be potential effectors. Compared to 0 hpi, 32 and 34 DEGs during C-00 and HKΔ4 infection were identified, respectively, which showed 25 shared, seven C-00 and nine HKΔ4 specific unigenes (Supplementary Fig. 4). Among the shared DEGs, 11 (44%) and 10 (40%) unigenes that showed the BCD at 6 and 12 hpi were identified in C-00 and HKΔ4 infection, respectively. However, the number of specific DEGs that showed the BCD at 20 hpi in the C-00 infection (5 unigene, 71.43% of the specific DEGs) was slightly bigger than the HKΔ4 infection (4 unigene, 44.44% of the specific DEGs) (Supplementary Fig. 4). The results demonstrate that the specific DEGs contributed for C-00 and HKΔ4 differential pathogenicity may not be related to their expression level.

### Number and expression patterns of the carbohydrate-active enzyme genes

CAZymes are involved in the metabolism of plant and fungal cell walls as well as in host-phytopathogen interactions^25^. Using the dbCAN database, a total of 600 CAZyme modules in 559 predicted proteins that are encoded by unigenes were identified. Among them, 360 unigenes responsible for cell wall degradation (CWD) were classified into 40 GHs, six CBMs, eight CEs, six PLs and nine AAs families, in which CBM42 and CBM43 occurred only in conjunction with GHs modules and were included in the GHs family (Supplementary Table 4). Of the 360 unigenes, 158 and 133 DEGs were identified during C-00 and HKΔ4 three infection time points compared to 0 hpi (Supplementary Table 4). The DEGs number for C-00 infection was reduced from 80 at 6 hpi to 70 at 12 hpi, and then increased to 114 at 20 hpi. For HKΔ4 infection, the DEGs number has been growing gradually from 6 to 20 hpi (Supplementary Fig. 5). There were noteworthy variation trends in the DEGs number of the plant CWD enzymes (cellulose, hemicellulose, pectin, and lignin degrading enzymes) and related CBM genes. For C-00 infection, the sum of DEGs at 6 and 12 hpi was much larger than the DEGs count at 20 hpi, but the results at 6 and 12 hpi showed fewer or equal DEGs compared to 20 hpi during the HKΔ4 infection, except cellulose degrading enzymes encoding genes (Supplementary Fig. 5).

Additionally, the expression patterns of the plant CWD enzymes and the related CBM encoding genes were further investigated. There were 58 shared, 48 C-00 and 26 HKΔ4 specific DEGs. In addition, 24 (41.38%) and 16 (27.59%) out of the 58 shared DEGs that exhibited the BCD at 6 and 12 hpi were found in C-00 and HKΔ4 infection, respectively, but 42 DEGs for HKΔ4 at 20 hpi showed the BCD, which was 13.79% more compared to C-00 infection (Supplementary Fig. 6). A similar trend existed for the specific DEGs: C-00 DEGs showed the BCD mainly at 6 and 12 hpi (52.08%), but HKΔ4 DEGs showed the BCD mainly at 20 hpi (61.54%) (Supplementary Fig. 6).

### Validation of the RNA-Seq data

**Figure 7:**
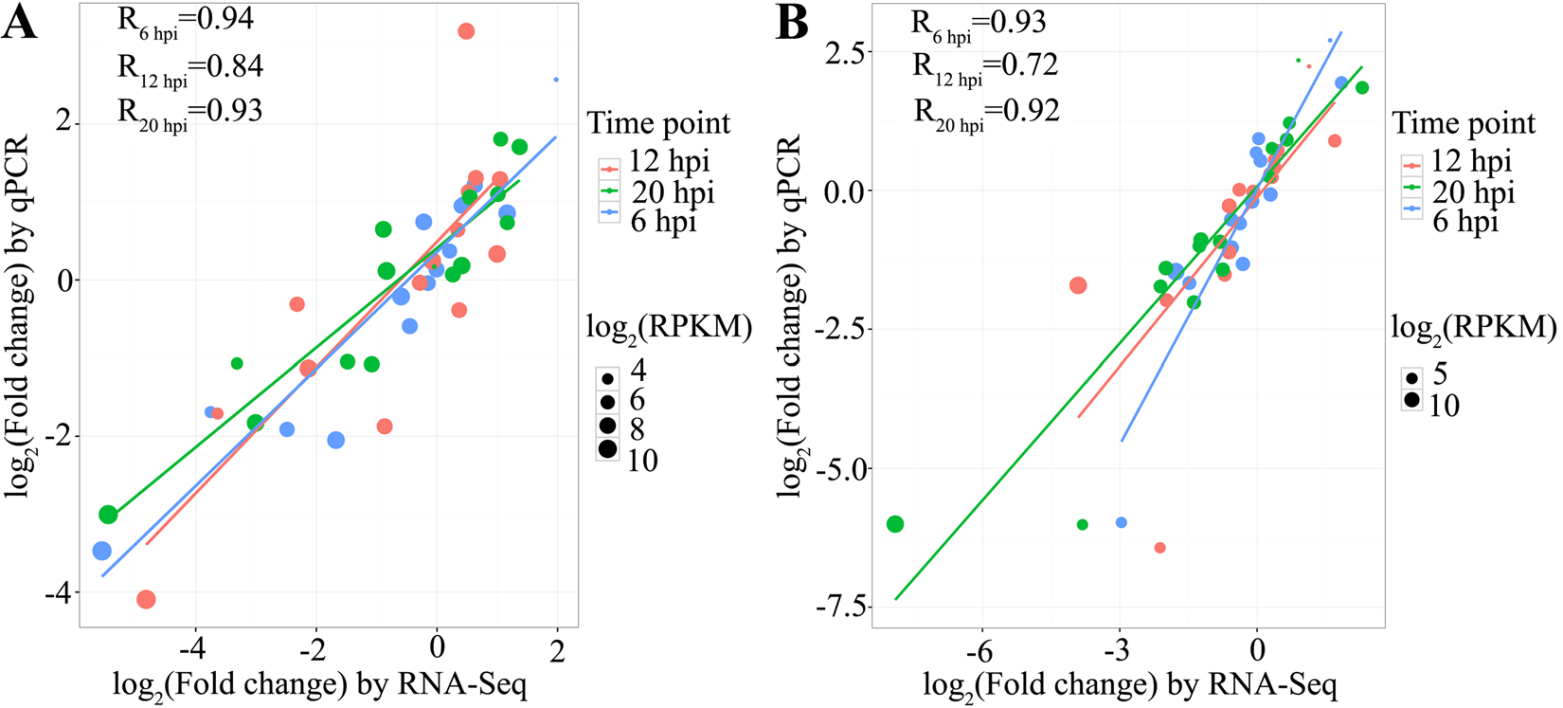
Correlation of gene expression ratios obtained by qPCR and RNA-Seq. (**A**) C-00 infection; (**B**) HKΔ4 infecion. Fold changes of the transcript level at three infection time points relative to 0 hpi are shown. X-axis and Y-axis represent the log_2_ value of the fold change from RNA-Seq and qPCR, respectively. The size of each point is proportional to the log_2_ (RPKM) at 0 hpi. All qPCR data are collected from three biological replicates.

To validate the RNA-Seq data, 15 genes (five CAZymes, three candidate effectors, two involved in secondary metabolites synthesis, three ABC transporters and two cytochrome P450s; Supplementary Table 5) were randomly selected for quantitative real-time PCR (qPCR) assays. A consistency between the RNA-Seq and qPCR results was found using correlation analysis. The Pearson correlation coefficient was 0.84–0.94 and 0.72–0.93 for C-00 and HKΔ4 infection, respectively (Fig. 7), which indicates further that the data produced through RNA-Seq is reliable. In addition, qPCR experiment was also performed to verify the interesting findings that two genes with antioxidant activity were specifically up-regulated in HKΔ4 (Supplementary Table 1). The result again showed that the qPCR result was highly consistent with the RNA-Seq data (Supplementary Fig. 7).

## Discussion

In our previous study, a group III HK from *A. longipes* (AlHK1) was proved to mediate pathogenicity in a negative manner^18^. To explore the molecular mechanism behind this phenomenon, two aspect experiments, confocal microscopy observation and time-course transcriptome analyses during *A. longipes* infection, were performed in this study. Confocal microscopy observation showed that the wild-type strain C-00 invaded faster than the *AlHK1*-disrupted strain HK∆4 in the early and middle infection stages, but in the late stage, HK∆4 caught up and overtook C-00, finally produced bigger lesion in tobacco leaves. To date, pathogenesis-associated genes negatively affected plant pathogenic fungi virulence were also reported elsewhere^15,26^, but none of them showed similar regulation pathogenic model to AlHK1.

From the time-course transcriptomes, unigenes encoding peptidases, transporters, cytochrome P450s and transcription factors were prevalent during *A. longipes* infection. Similar results were reported in some other phytopathogenic fungi infection processes^27,28^. The relationship between peptidases, transporters, cytochrome P450s, transcription factors and pathogen virulence has been widely determined. More specifically, peptidases may degrade plant cell wall proteins to facilitate fungal hyphal penetration or may degrade plant defense proteins^29^. The transporters, a class of integral membrane proteins, may play important roles in pathogenic fungal pathogenesis, either by acquiring the nutrients of their hosts to support their growth or by exporting (i) compounds involved in pathogenesis such as secondary metabolites and (ii) host-derived antimicrobial compounds^30^. For example, Wahl *et al*. found that a sucrose transporter was required for plant pathogen *Ustilago maydis* virulence^31^. Cytochrome P450s are involved in detoxification of host toxins and allow pathogenic fungi to grow under different conditions^32^. Some transcription factors were proved to regulate *A. brassicicola* pathogenesis^14–16^. Therefore, these prevalent genes may play conserved roles on pathogenic fungal pathogenesis regardless of infection stages and fungal species.

In general, plant cells can rapidly generate large amounts of ROS in an oxidative burst as early defence reactions against pathogen attack^33^. To contradict the hosts, necrotrophic pathogens also produce ROS themselves to induce plant tissue necrosis and facilitate infection^34^. Therefore, pathogens must be able to overcome the ROS-mediated oxidative stress during their infection. Indeed, pathogenic fungi have evolved many strategies, including enzymatic and non-enzymatic systems, to scavenge ROS^35^. However, no previous studies have reported the doses of oxidative stress that the fungi can resist during infection. In this study, genes for four catalases, two peroxidases and one peroxiredoxin that have ability to alleviate the damage caused by ROS were found only in the HKΔ4 DEGs, suggesting they may be regulated by AlHK1. As a result, HKΔ4 is much more resistant to low concentrations H_2_O_2_ and menadione than C-00 (Fig. 4). The results indicate the different abilities of coping with oxidative stress between C-00 and HK∆4 conferred by some unigenes with antioxidant activity may be a major reason for the differences in virulence at lesion-forming stage. Likewise, the fact that two-component HK regulates the expression of some core oxidative stress response genes (e.g., genes for catalases, thioredoxin reductases and glutathione S-transferases) was also reported by another research group^36^.

Since the genomes of C-00 and HKΔ4 are different in only one gene (*AlHK1*), their differential infection characteristics should be modulated by AlHK1, and it can be viewed from two aspects: DEGs counts and their expression patterns. The statistics for the ratio of DEGs and for the number of DEGs between two adjacent time points, GO enriched terms, DEGs in clusters with rising trends, and plant CWD enzymes-encoding genes gives a firm conclusion: changes of DEGs number in C-00 infection happened mainly in the early and middle stages, while that in HKΔ4 infection mainly at the late stage. Theoretically, the expression patterns of the DEGs, even containing the C-00 and HK∆4 shared DEGs, may be related to AlHK1. Actually, the time-course expression abundance of the prevalent shared DEGs in C-00 infection was different from that in HK∆4 infection and a conclusion similar to the statistics analysis of DEGs counts was drew. The conclusion was supported by a similar findings of Cho *et al*. that two hydrolytic enzyme genes of encoding lipase and cellobiohydrolase were not differentially expressed in the early infection stage, whereas their transcript levels were higher in a transcription factor mutant than in the wild type strain in the late infection stage^15^. The conclusion was also further supported by the expression pattern analysis of (i) the shared DEGs of the secondary metabolite biosynthesis, (ii) the secreted proteins encoding genes, (iii) the shared DEGs of the candidate effectors, and (iv) the CAZymes encoding genes. In fungi, HK signalling has been implicated in the regulation of secondary metabolite biosynthesis^37^ and some of secondary metabolites are involved in fungal pathogens pathogenesis^2^. To our knowledge, the involvement of two-component HK in the regulation of expression of secreted proteins, effectors and CAZymes in fungal pathogens has not been reported as yet. However, the possibility of their expressions modulated by AlHK1 was not excluded. Actually, a two-component response regulator Bbssk1, which is thought to be downstream component of HK, was discovered to regulate the expression of some effector, GH and GT encoding genes^38^.

Undoubtedly, omostic stress is a type of common stress during pathogens infection. In this study, C-00 has more up-regulated and less down-regulated osmoresponsive genes than HK∆4 in the early infection stage, whereas HK∆4 inverses the condition in the late stage (Fig. 6). The data suggests that, in the regulation of AlHK1, HK∆4 delay adaptation to the osmotic stress during infection, which make C-00 invade faster in the early infection stages (Fig. 1).

In summary, the results from the confocal microscopy observation and time-course transcriptome analyses reveal that changes of infection phenotype, DEGs counts and gene expression level in the C-00 infection happened mainly in the early and middle stages, while HKΔ4 switches the situation in the late infection stage. In another word, our data supports the conclusion that AlHK1 may modulates *A. longipes* virulence through a come-from-behind mechanism and provide a robust basis for further dissection of genes relevant to antioxidant activity, osmoadaption and virulence, etc. in this necrotrophic phytopathogen.

## Methods

### Biological materials and media

The wild-type strain C-00 and the *AlHK1*-disrupted strain HKΔ4 constructed in our previous study^18^ were grown on potato dextrose agar (PDA; HKM, Guangzhou, China). Tobacco leaves from a susceptible cultivar (*Nicotiana tabacum* K326) were used for *A. longipes* pathogenicity assays.

### Pathogenicity assays

The infection experiment was carried out according to our previous study^18^ with a minor modification. Briefly, healthy fresh leaves were harvested from the bottom of 6-week-old tobacco plants and surfaces were disinfected with 1% (m/v) NaClO for 2 min and rinsed with sterile ddH_2_O four times. The pre-treated tobacco leaves were placed into sterile petri dishes that contained two pieces of blotting paper at the bottom. Mycelial plugs with a 10-mm diameter were inoculated on the surface of tobacco leaves and 2 ml sterile ddH_2_O was added to the petri dishes to increase humidity and facilitate infection. Symptoms were developed at 28 °C for 3 days using a 12-h light/12-h dark photoperiod and were recorded daily through photography. The pathogenicity assay for each strain was repeated three times using at least five leaves per assay.

### Confocal microscopy

Similar to the report of Sun *et al*.^39^, fungal hyphae and plant cells were stained, respectively, with the fluorescent dyes WGA-CF488A (Biotium, Bay aera, CA) and propidium iodide (Sigma-Aldrich, Darmstadt, Germany). Infected plant tissues where mycelial plugs (10-mm in diameter) located from 0, 2, 4, 6, 10, 12, 16, 20, 24, 36 hpi were trimmed with scissors and mycelial plugs were removed by a pair of forceps. After successive treatments with 100% (v/v) EtOH and 10% (m/v) NaOH, the plant tissues were incubated in staining solution [20 μg/ml propidium iodide, 10 μg/ml WGA-CF488A, 0.02% (v/v) Tween 20, 10 mM phosphate buffered saline (PBS) (pH 7.4)] for 30 min and subsequently washed in PBS (pH 7.4). Confocal images were acquired using a FV1000 laser scanning confocal microscope (Olympus, Tokyo, Japan). For WGA-CF488A fluorescence, there was an excitation of 488 nm and detection occurred at 490–530 nm. Excitation at 561 nm and detection at 600–700 nm were used for propidium iodide visualization.

### RNA extraction, library construction and sequencing

In the pathogenicity assays, mycelial plugs from 0, 6, 12 and 20 hpi were pulled out. In ensuring the integrity of plant tissues of the premise, fungal hyphae were obtained by scraping with sterile inoculating needle from the infected surfaces of fungal plugs and tobacco leaves. The fungal hyphae were gathered from several scrape and used for RNA extraction. The total RNA was isolated using the Trizol Kit (Promega, Madison, WI) following by the manufacturer’s protocol and treated with RNase-free DNase I (Takara, Dalian, China) to remove genomic DNA contamination. In total, eight RNA samples (each sample mixed with 2–3 aliquots RNA, wherein each aliquot was extracted with 12–15 hyphae from 4–5 tobacco leaves) were subjected to RNA-Seq library construction using an Illumina TruSeq RNA Sample Preparation Kit (Illumina, San Diego, CA) following the manufacturer’s instructions. Then, the RNA-Seq library was sequenced using Illumina HiSeq™ 2500 (Illumina, San Diego, CA) as 100 nt paired-end raw reads.

### Transcriptome assembly and annotation

A Perl program was written to select clean reads by removing reads including adapter sequencing or empty adapter, reads with more than 5% N bases and low-quality reads with base quality < 20 exceeding 50% in one sequence. The clean reads were used for *de novo* assembly with Trinity^40^ and Oases^41^. All of the assembled transcripts were defined as unigenes. To obtain protein functional annotation, unigenes were used for BLASTx alignment (E-value of <1e^−5^) against the Nr, Swiss-Prot, COG and KEGG databases. With Nr annotation, the Blast2GO platform^42^ was used to obtain GO annotation of the unigenes. Then, the GO terms were analysed with WEGO programme^43^ to classify the unigenes into biological process, cellular component and molecular function.

### Analysis of differentially expressed genes

Aligned reads were quantified using the RPKM method^44^. DEGs between two samples were identified according to the restrictive conditions of an absolute value of log_2_ fold changes in RPKM values ≥ 1 and false discovery rate (FDR) ≤ 0.001. As for the unigenes that are expressed in one sample but not express in another, a minimal RPKM value (0.001) was assigned to facilitate the fold change calculation. The DEGs were then subjected to GO according to a method similar to the one described by Xue *et al*.^45^, and corrected *P* values ≤ 0.05 were considered to be significantly enriched in DEGs. Heatmaps of gene expression profiles were generated using R package (v3.1.3; http://www.R-project.org/). To investigate the dynamic patterns of the DEGs during the infection process, gene expression cluster analysis was performed using the STEM (v1.3.8) algorithms^46^.

### Sensitivity testing to oxidative stress *in vitro*

The sensitivity testing was determined using hyphal growth assay. The PDA plates supplemented with 1, 10, 20, 30 mM H_2_O_2_ and 0.01, 0.05, 0.1 mM menadione were inoculated with mycelial plugs of a 6-mm diameter and incubated at 28 °C for 5 days. Colony diameters were measured and all experiments were repeated three times with 4–5 plates for each treatment. RGI was calculated as described in our previous study^47^. Data comparison between two groups was analysed using Student’s t-test.

### Prediction of genes involved in fungal pathogen virulence

For the prediction of the secondary metabolite biosynthesis genes, a web-based software SMURF (http://jcvi.org/smurf/run_smurf.php) was used. The secreted proteins were obtained through several prediction algorithms. SignalP (v3.0), TargetP (v1.1b) and TMHMM (v2.0c) (http://www.cbs.dtu.dk/services/) were used to detect protein sequences with signal peptides, protein subcellular location and protein transmembrane helices, respectively. Proteins with signal peptides, lacking transmembrane domains and not located in mitochondrion were considered to be secreted proteins. In general, effectors are small secreted cysteine-rich proteins^6^. The secreted proteins with a length of less than 200 amino acids and with a cysteine content of at least 1.5% were deemed candidate effectors. Some unigenes without intact transcripts were removed from the candidate effectors even though they meet the conditions for effector screening. The putative CAZymes were identified using the HMMScan programme against the dbCAN database^48^ and strict screening conditions with an E-value of <1e^−3^ for alignments of <80 amino acids and an E-value of <1e^−5^ for alignments of ≥80 amino acids as well as an alignment covered fraction >0.5 were used. The sub-classification of CAZymes was carried out according to the methods of Blackman *et al*.^49^.

### qPCR validation

RNA was extracted as a different set of biological samples under the same conditions as those used in RNA-Seq. Three independent biological replicates were performed for each sample. The extracted RNA was used to synthesize cDNA using a Hiscript II Q RT SuperMix for qPCR (+gDNA wiper) Kit (Vazyme, Nanjing, China) according to the manufacturer’s instructions. qPCR was performed using the ABI Prism 7500 sequence detection system (Perkin-Elmer, Waltham, MA) with SYBR Green I fluorescent dye detection (Vazyme, Nanjing, China). A house-keeping gene *act* encoding for the actin protein was chosen as the endogenous control. The primers used for qPCR are listed in Supplementary Table 5 and a 2^−ΔCt^ method^50^ was used to analyse the relative changes in gene expression.

### Data availability

The RNA-Seq raw data were deposited in the NCBI Sequence Read Archive (SRA) under accession number SRP094534.

## Electronic supplementary material

Supplementary Information

Supplementary Table 1

Supplementary Table 2

Supplementary Table 3

Supplementary Table 4

Supplementary Table 5
