# Supplementary material for: Transcriptomic profiling of Alternaria longipes invasion in tobacco reveals pathogenesis regulated by AlHK1, a group III histidine kinase
Source: Sci Rep. 2017 Nov 22;7:16083. doi: 10.1038/s41598-017-16401-6 (PMC5700128; doi:10.1038/s41598-017-16401-6)
Supplement: Supplementary file 1 — Supplementary Information [file 41598_2017_16401_MOESM1_ESM.pdf]

# Transcriptomic profiling of *Alternaria longipes* invasion in tobacco reveals pathogenesis regulated by AlHK1, a group III histidine kinase

Juan Yang, Zhi-Qun Yin, Zi-Teng Kang, Chen-Jian Liu, Jin-Kui Yang, Jian-Hua Yao and Yi-Yong Luo

**Supplementary Table 1. Shared and specific differentially expressed genes during *Alternaria longipes* infection.**

**Supplementary Table 2. Gene ontology enrichment analysis of differentially expressed genes during *Alternaria longipes* infection.**

**Supplementary Table 3. Prediction of the secondary metabolite biosynthesis genes.**

**Supplementary Table 4. Annotation and differentially expressed gene identification of the carbohydrate-active enzyme families associated with cell wall degradation in the *Alternaria longipes* invasion transcriptome.**

**Supplementary Table 5. Primer sequences used for quantitative real-time PCR analysis.**

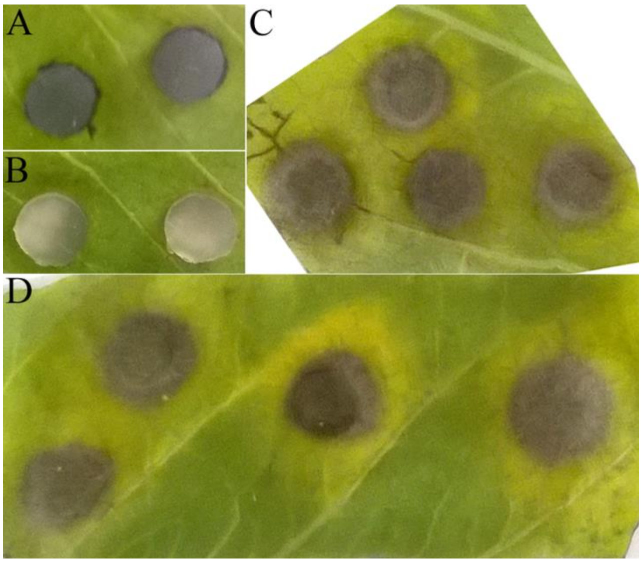


**Supplementary Figure 1. Pathogenicity assays.** Detached tobacco leaves were inoculated with mycelial plugs from C-00 (**A** and **C**) and HKΔ4 (**B** and **D**), and then they were incubated at 28 ℃ for 1 h (**A** and **B**) or 3 days (**C** and **D**). Only representative replicates are shown.

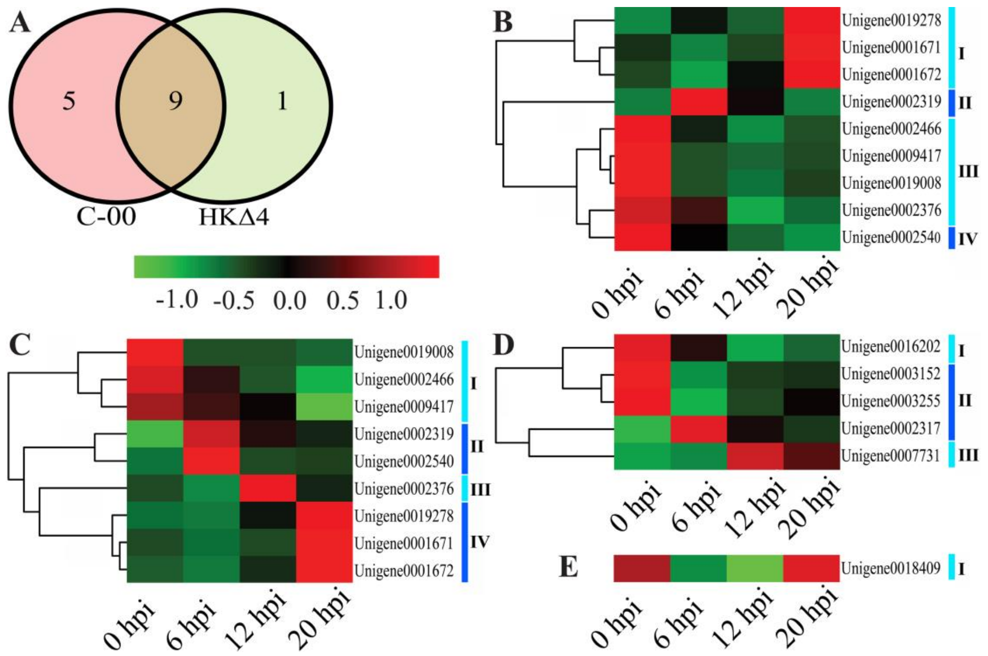


**Supplementary Figure 2. The number and expression patterns of differentially expressed genes (DEGs) involved in secondary metabolite biosynthesis.** The colours correspond to the value of RPKM, which range from green (low expression) to red (high expression). (**A**) The Venn diagram showing the number of shared and specific DEGs between C-00 and HKΔ4. (**B**) Expression patterns of the shared DEGs in the C-00 infection. DEGs in cluster II, cluster III and clusters I, IV show the biggest change degree (BCD) of gene expression level at 6, 12 and 20 hpi, respectively. (**C**) Expression patterns of the shared DEGs in the HKΔ4 infection. DEGs in cluster II, cluster III and clusters I, IV show the BCD at 6, 12 and 20 hpi, respectively. (**D**) Expression patterns of C-00 specific DEGs. DEGs in cluster II and clusters I, III show the BCD at 6 and 12 hpi, respectively. (**E**) Expression patterns of HKΔ4 specific DEGs. DEGs in cluster I show the BCD at 12 hpi, respectively.

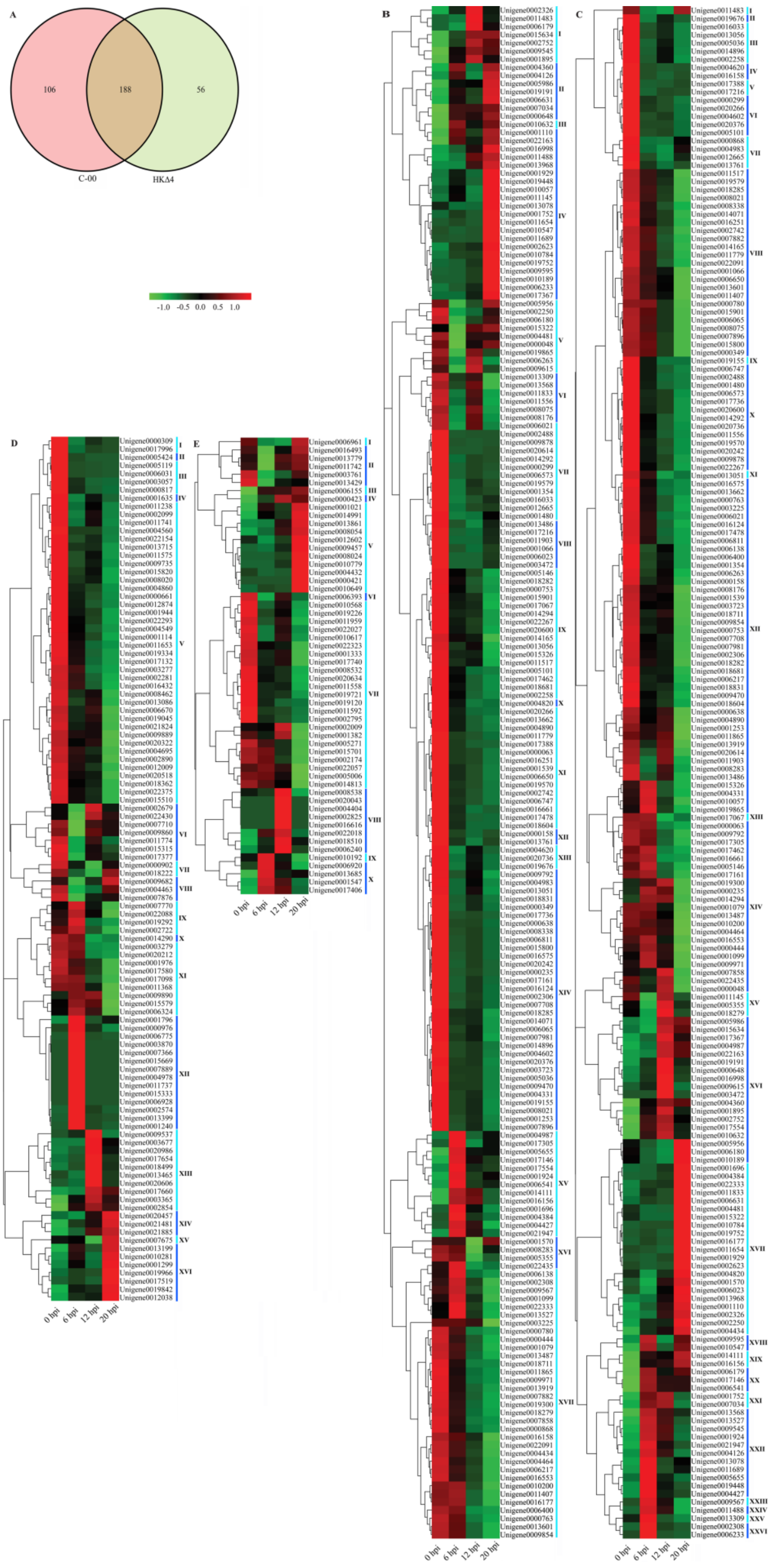


**Supplementary Figure 3. The number and expression patterns of differentially expressed genes (DEGs) encoding secreted proteins.** The colours correspond to the

value of RPKM, and they range from green (low expression) to red (high expression). (**A**) The Venn diagram showing the number of shared and specific DEGs between C-00 and HKΔ4. (**B**) Expression patterns of the shared DEGs in the C-00 infection. DEGs in clusters III, V, VII, XIII, XV, clusters I, VIII, X, XII, XVI and clusters II, IV, VI, IX, XI, XIV, XVII show the biggest change degree (BCD) of gene expression level at 6, 12 and 20 hpi, respectively. (**C**) Expression patterns of the shared DEGs in the HKΔ4 infection. DEGs in clusters II, V, XV, XVIII, XX, XXII, XXIV, XXVI, clusters I, IV, VII, IX, XI, XIII, XVI, XXI and clusters III, VI, VIII, X, XII, XIV, XVII, XIX, XXIII, XXV show the BCD at 6, 12 and 20 hpi, respectively. (**D**) Expression patterns of C-00 specific DEGs. DEGs in clusters I, IV, VI, VIII, XII, clusters II, VII, X, XIII, XV and clusters III, V, IX, XI, XIV, XVI show the BCD at 6, 12 and 20 hpi, respectively. (**E**) Expression patterns of HKΔ4 specific DEGs. DEGs in clusters II, VI, X, clusters I, IV, VIII and clusters III, V, VII, IX show the BCD at 6, 12 and 20 hpi, respectively.

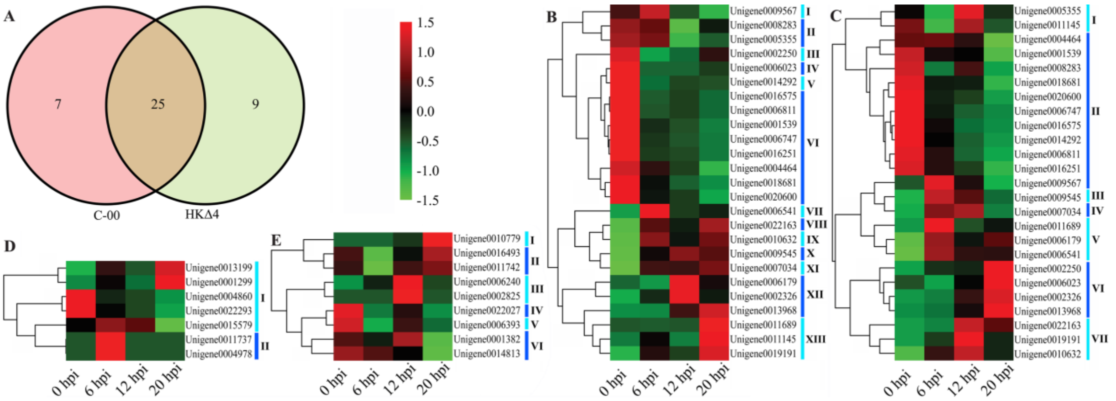


**Supplementary Figure 4. The number and expression patterns of differentially expressed genes (DEGs) encoding candidate effectors.** Green, black and red indicate low, no and high expression, respectively. (**A**) The Venn diagram showing the number of shared and specific DEGs between C-00 and HKΔ4. (**B**) Expression patterns of the shared DEGs in the C-00 infection. DEGs in clusters III, V, VII, IX, clusters II, IV, X, XII and clusters I, VI, VIII, XI, XIII show the biggest change degree (BCD) of gene expression level at 6, 12 and 20 hpi, respectively. (**C**) Expression patterns of the shared DEGs in the HKΔ4 infection. DEGs in clusters I, III, V, clusters IV, VII and clusters II, VI show the BCD at 6, 12 and 20 hpi, respectively. (**D**) Expression patterns of C-00 specific DEGs. DEGs in cluster II and cluster I show the BCD at 6 and 20 hpi, respectively. (**E**) Expression patterns of HKΔ4 specific DEGs. DEGs in clusters II, V, cluster III and clusters I, IV, VI show the BCD at 6, 12 and 20 hpi, respectively.

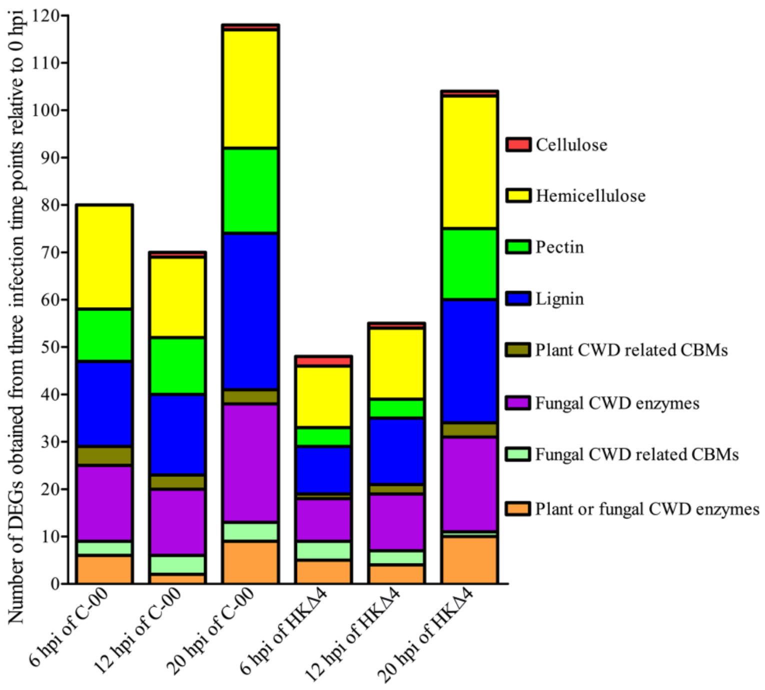


**Supplementary Figure 5. Number of cell wall degradation (CWD) enzymes and CWD-related carbohydrate-binding modules.** The data are from Supplementary Table 4.

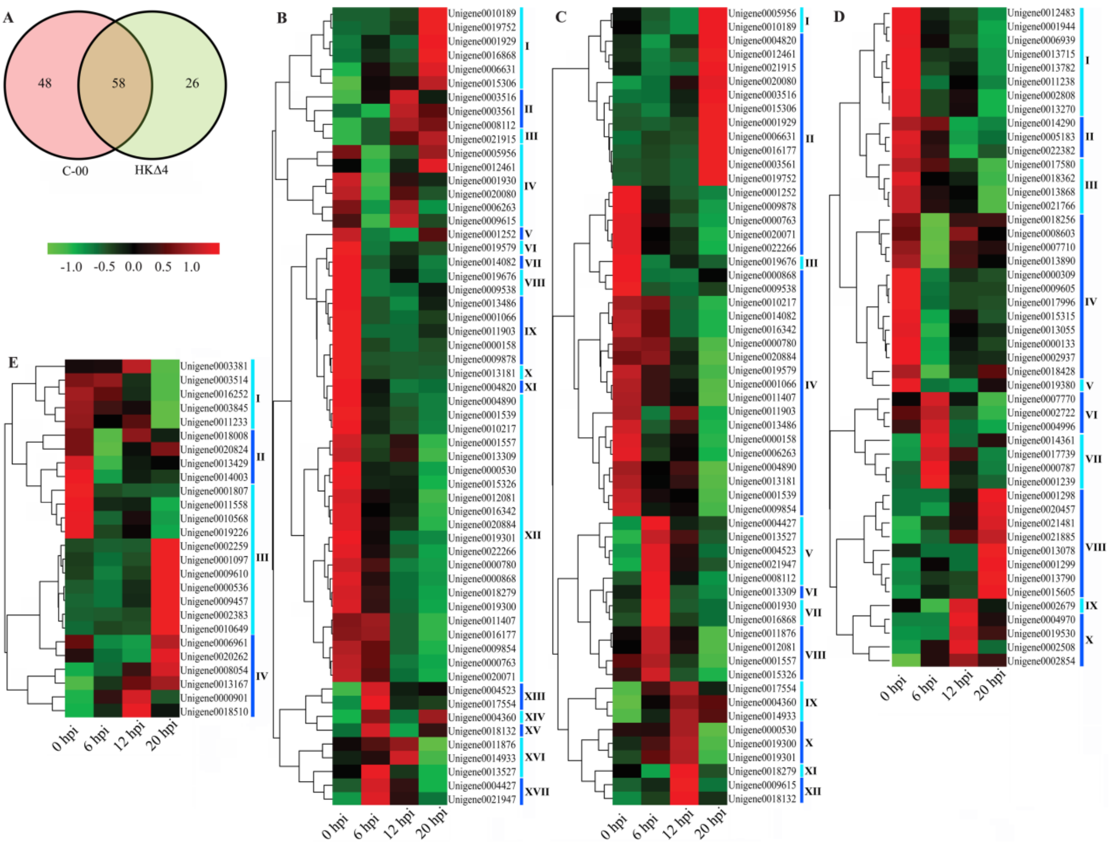


**Supplementary Figure 6. The number and expression patterns of differentially expressed genes (DEGs) encoding plant cell wall degradation enzymes and related carbohydrate-binding modules.** Green, black and red indicate low, no and high expression, respectively. (**A**) The Venn diagram showing the number of shared and specific DEGs between C-00 and HKΔ4. (**B**) Expression patterns of the shared DEGs in the C-00 infection. DEGs in clusters IV, VI, VIII, VIII, XV, XVII, clusters II, V, IX, XI and clusters I, III, VII, X, XII, XIV, XVI show the biggest change degree (BCD) of gene expression level at 6, 12 and 20 hpi, respectively. (**C**) Expression patterns of the shared DEGs in the HKΔ4 infection. DEGs in clusters III, V, VII, XI, clusters I, IX, XII and clusters II, IV, VI, VIII, X show the BCD at 6, 12 and 20 hpi, respectively. (**D**) Expression patterns of C-00 specific DEGs. DEGs in clusters IV, VII, IX, clusters II, V, X and clusters I, III, VI, VIII show the BCD at 6, 12 and 20 hpi,

respectively. (**E**) Expression patterns of HKΔ4 specific DEGs. DEGs in cluster II, cluster IV and clusters I, III show the BCD at 6, 12 and 20 hpi, respectively.

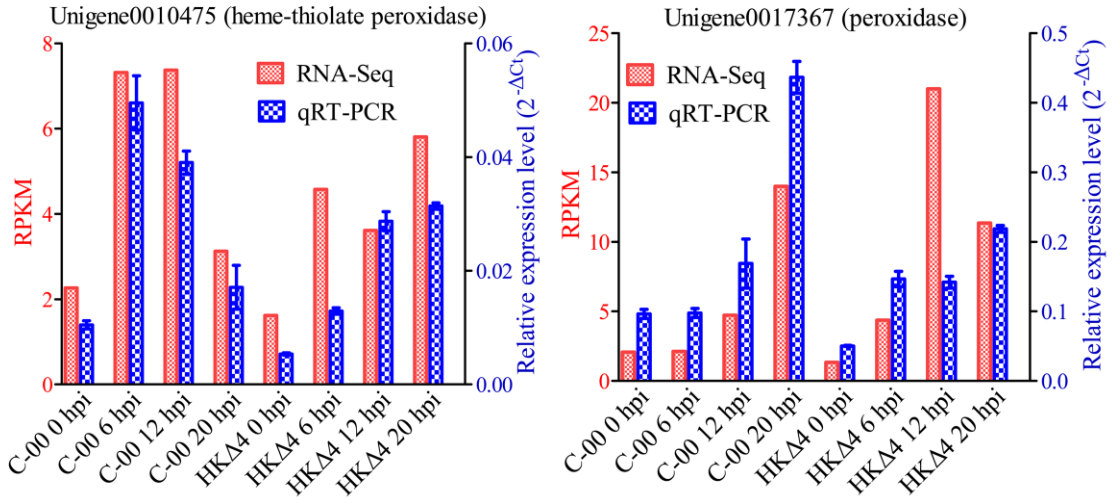


**Supplementary Figure 7. The correlation of two antioxidant activity genes being specifically up-regulated in HKΔ4 between RNA-Seq and qRT-PCR analyses.** The *actin* gene was chosen as internal control and the relative gene expression levels of two genes were determined by RNA-Seq (Red) and qRT-PCR (Blue). Error bars show standard deviations (SD) from three repeated experiments.
